# Cooperative role of LSD1 and CHD7 in regulating differentiation of mouse embryonic stem cells

**DOI:** 10.1038/s41598-024-78920-3

**Published:** 2024-11-18

**Authors:** Sandhya Malla, Carlos Martinez-Gamero, Kanchan Kumari, Cyrinne Achour, Georgios Mermelekas, David Martinez-Delgado, Alba Coego, Diana Guallar, Angel-Carlos Roman, Francesca Aguilo

**Affiliations:** 1 Department of Molecular Biology, Umeå University, 901 85 Umeå, Sweden; 2 Wallenberg Centre for Molecular Medicine, Umeå University, 901 85 Umeå, Sweden; 3 Science for Life Laboratory, Department of Oncology-Pathology, Karolinska Institutet, 171 21 Solna, Sweden; 4 Center for Research in Molecular Medicine and Chronic Diseases (CIMUS), Universidade de Santiago de Compostela (USC)-Health Research Institute (IDIS), Santiago de Compostela, Spain; 5 Department of Biochemistry, Molecular Biology and Genetics, University of Extremadura, Badajoz, Spain

**Keywords:** Embryonic stem cells, Molecular biology, Stem cells

## Abstract

Lysine-specific histone demethylase 1 (LSD1) is a histone demethylase that plays a critical role in epigenetic regulation by removing the methyl group from mono- and di-methylated lysine 4 on histone H3 (H3K4me1/2), acting as a repressor of gene expression. Recently, catalytically independent functions of LSD1, serving as a scaffold for assembling chromatin-regulator and transcription factor complexes, have been identified. Herein, we show for the first time that LSD1 interacts with chromodomain-helicase-DNA-binding protein 7 (CHD7) in mouse embryonic stem cells (ESCs). To further investigate the CHD7–LSD1 crosstalk*,* we engineered *Chd7* and *Chd7/Lsd1* knockout (KO) mouse ESCs. We show that CHD7 is dispensable for ESC self-renewal and survival, while *Chd7* KO ESCs can differentiate towards embryoid bodies (EBs) with defective expression of ectodermal markers. Intriguingly, *Chd7/Lsd1* double KO mouse ESCs exhibit proliferation defects similar to *Lsd1* KO ESCs and have lost the capacity to differentiate properly. Furthermore, the increased co-occupancy of H3K4me1 and CHD7 on chromatin following *Lsd1* deletion suggests that LSD1 is required for facilitating the proper binding of CHD7 to chromatin and regulating differentiation. Collectively, our results suggest that LSD1 and CHD7 work in concert to modulate gene expression and influence proper cell fate determination.

**Supplementary Information:**

The online version contains supplementary material available at 10.1038/s41598-024-78920-3.

## Introduction

Embryonic stem cells (ESCs) are derived from the inner cell mass (ICM) of pre-implantation embryos. ESCs are pluripotent i.e., they have the capacity of unlimited self-renewal, and the potential to differentiate into a wide range of mature specialized cells from the three embryonic germ layers. Self-renewal and pluripotency are tightly regulated by the core regulatory circuitry of pluripotency factors, consisting of OCT4, NANOG, and SOX2^1,2^. Besides specific transcriptional regulation, epigenetic modifications also contribute to the maintenance of stemness, characterized by an open chromatin structure compared with somatic cells. Another characteristic of ESCs is the poised bivalent state of developmental regulatory genes that harbor both activating [histone H3-lysine 4 trimethylation (H3K4me3)] and repressive [(histone H3-lysine 27 trimethylation (H3K27me3)] histone marks^3^. These developmental genes are silenced in ESCs, but the so-called bivalent domains allow their rapid activation upon induction of differentiation.

As an epigenetic regulator, lysine-specific histone demethylase 1 (LSD1), also known as KDM1A or AOF2 or BHC110, plays pleiotropic cellular functions in ESC self-renewal and pluripotency^4^. LSD1 is a flavin-dependent demethylase that mainly catalyzes the demethylation of the mono- and di-methyl moieties from H3K4 (H3K4me1/2), and thus functions as a transcriptional repressor^5,6^. The mechanism by which LSD1 regulates transcription remains unclear due to its network of interactors, which are target-specific. For instance, LSD1 has been identified in CoREST^6,7^, CtBP^8^, androgen receptor-CHD1^9^, HDAC-containing NuRD and Sin3a complexes^10,11^, and it has also been shown to interact with the transcription factors ZNF217^12^ and SNAIL1/2^13^. Intriguingly, it has been reported that CoREST regulates breast tumorigenesis independently of LSD1 enzymatic activity. Whether LSD1 acts as a scaffold protein in all aforementioned complexes and all cellular contexts remains to be elucidated^14,15^.

Chromatin remodeling proteins regulate bivalent chromatin, allowing a transition between an activated or a silenced state of gene expression, which is fundamental to maintaining pluripotency and undergoing differentiation^16^. Chromodomain-helicase DNA binding (CHD) proteins are chromatin remodeler proteins that read histone marks and facilitate nucleosome mobilization through their SNF2-like ATP-dependent helicase domain^17,18^. CHD7, a member of the CHD family of proteins, is crucial for cell fate decisions. CHD7 binds at active enhancers decorated with H3K4me1 to regulate the expression of pluripotency genes^19,20^. Although ESC self-renewal is not dependent on CHD7 function, CHD7 plays a central role in ectodermal differentiation and neural crest induction^21^. Furthermore, CHD7 facilitates the establishment of an open chromatin state at the promoters of its target genes during neurogenesis^22^. Notably, autosomal dominant mutations in *CHD7* are the leading cause of the CHARGE syndrome^23,24^, a rare genetic disorder that affects multiple organ systems.

In this study, we have identified CHD7 as a novel LSD1-interacting partner using an LC–MS/MS-based targeted proteomics approach. Interestingly, we found enhanced binding of CHD7 to chromatin upon loss of *Lsd1* in mouse ESCs. Through molecular and phenotypic characterization of *Chd7* and *Chd7/Lsd1* knockout (KO) mouse ESCs, we demonstrated that CHD7 is not required for ESC self-renewal but for proper induction of ectodermal markers. Similar to *Lsd1* KO mouse ESCs, *Chd7/Lsd1* double KO mouse ESCs displayed proliferation and differentiation defects. Taken together, our results suggest that LSD1 and CHD7 plays a central role in mediating the interplay of distinct epigenetic signatures during cell fate decisions.

## Results

### CHD7 is a novel interactor of LSD1

**Fig. 1:**
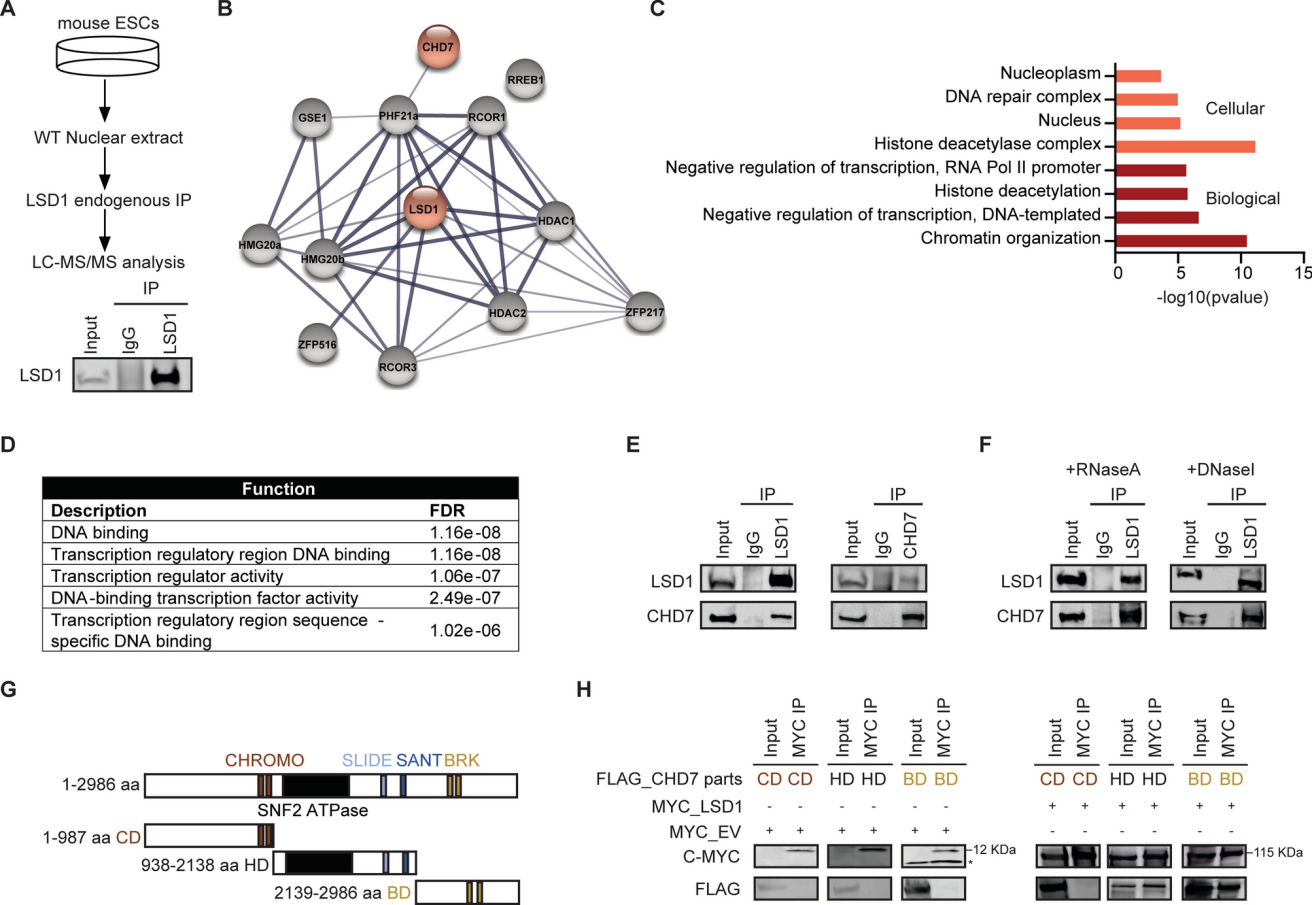
CHD7 interacts with LSD1 in ESCs. (**A**) Scheme representing the workflow for LSD1 immunoprecipitation (IP) and western blot (WB) of LSD1-IP depicting the specificity of LSD1 antibody. IgG was used as a negative control. Input corresponds to 10% of the nuclear extract used for IP. (**B**) STRING network of LSD1-interacting proteins retrieved from the LC–MS/MS analysis that followed LSD1-IP. Line thickness indicates the strength of data support. (**C**) GO analysis for cellular components and biological processes of top LSD1-interacting candidates. (**D**) Molecular functions of the top 13 interacting candidates of LSD1. (**E**) IP of LSD1 (left panel) and CHD7 (right panel) from nuclear extracts of mouse ESCs followed by WB with anti-LSD1 and anti-CHD7 antibodies. IgG was used as a negative control. Input corresponds to 10% of the nuclear extract used for IP. The represented blots are from different gels of the same biological replicate. (**F**) IP of LSD1 from nuclear extracts of mouse ESCs pretreated with RNase A (left panel) or DNase I (right panel), followed by WB with anti-LSD1 and anti-CHD7 antibodies. IgG was used as a negative control. Input represents 10% of the nuclear extract used for IP. The represented blots are from different gels of the same biological replicate. (**G**) Schematic representations of the different domains of CHD7 used for cloning in pCMV8-Flag tagged plasmids. (**H**) IP of c-MYC from nuclear extracts of HEK293T co-transfected with the indicated constructs from (G) and pSIN-c-MYC containing LSD1 followed by c-MYC and FLAG WB. IgG was used as a negative control. The represented blots are from the same gel of the same biological replicate. Results are one representative of n = 3 independent biological experiments (**A**, **E**, **F** and **H**) and n = 2 (**B**–**D**).

To gain deeper insights into the role of LSD1 in stem cell biology, we performed immunoprecipitation (IP) of endogenous LSD1 using an anti-LSD1 antibody, followed by LC–MS/MS analysis (Fig. 1A and B). Thirteen high-confidence LSD1-interacting proteins meeting the inclusion criteria of fold change > 2 and *P* < 0.001 were identified (Table S1). We retrieved known interactors of LSD1, such as HDAC-containing NuRD and Sin3a complexes^8^, ZNF217^25^, the CoREST/REST corepressor 1 and 3 (RCOR1/3)^26,27^, thereby confirming the efficacy of our approach (Fig. 1B). Additionally, our analysis revealed a novel interaction between LSD1 and CHD7, an ATP-dependent chromatin remodeler, which has not been previously reported. Gene ontology (GO) terms associated with the biological process and cellular component categories of the thirteen LSD1 interactors revealed categories related to “chromatin organization”, “transcription”, “DNA repair”, and “histone deacetylase” (Fig. 1C). Moreover, molecular function-based GO terms were highly enriched in categories related to “DNA binding” and “transcription regulation” (Fig. 1D), highlighting the central role of LSD1 and its interacting proteins in epigenetic regulation.

We further investigated the interaction of LSD1 with CHD7 since: (i) this interaction is novel; (ii) similar to LSD1^28^, CHD7 binds at enhancers of pluripotency genes^19^; and (iii) the binding of CHD7 to DNA is influenced by H3K4me1, a histone mark that is targeted by LSD1^19^. First, we verified the association between LSD1 and CHD7 in mouse ESCs by performing co-immunoprecipitation (co-IP) (Fig. 1E; *left panel*) and reverse co-IP experiments of the endogenous proteins (Fig. 1E; *right panel*). Notably, LSD1–CHD7 interaction was DNA and RNA-independent, as treatment of the nuclear extracts with DNase I or RNase A, respectively, did not disrupt the interaction of the two proteins (Fig. 1F).

CHD7 contains several multi-functional domains, including two N-terminal chromodomains (CD) which are required for histone mark recognition, a SWI2/SNF2-like ATPase/helicase domain (HD), a SANT domain that enhances nucleosome remodeling efficiency, and two BRK domains (BD) with uncharacterized functions. To pinpoint the specific region of CHD7 responsible for the interaction with LSD1, we engineered distinct pCMV8-FLAG-tagged constructs to express the different domains of CHD7 (Fig. 1G): (i) construct CD, encompassing the N-terminal fraction with the two CD domains; (ii) construct HD, representing the central region of CHD7 with the ATPase/helicase, SLIDE and SANT domains; and iii) the C-terminal domain with the BRK domains. The aforementioned constructs were transiently co-expressed with pSIN-c-MYC tagged full-length LSD1 in HEK293T cells. CHD7 and LSD1 interactions were next assessed by FLAG-immunoprecipitation of nuclear extracts, followed by western blotting against FLAG and MYC. Our results showed that LSD1 interacted with CHD7 through its ATPase/helicase domain and BRK domain (Fig. 1H), suggesting that LSD1 can modulate the nucleosome remodeling function of CHD7.

### CHD7 colocalizes with LSD1 at distal regulatory regions

**Fig. 2:**
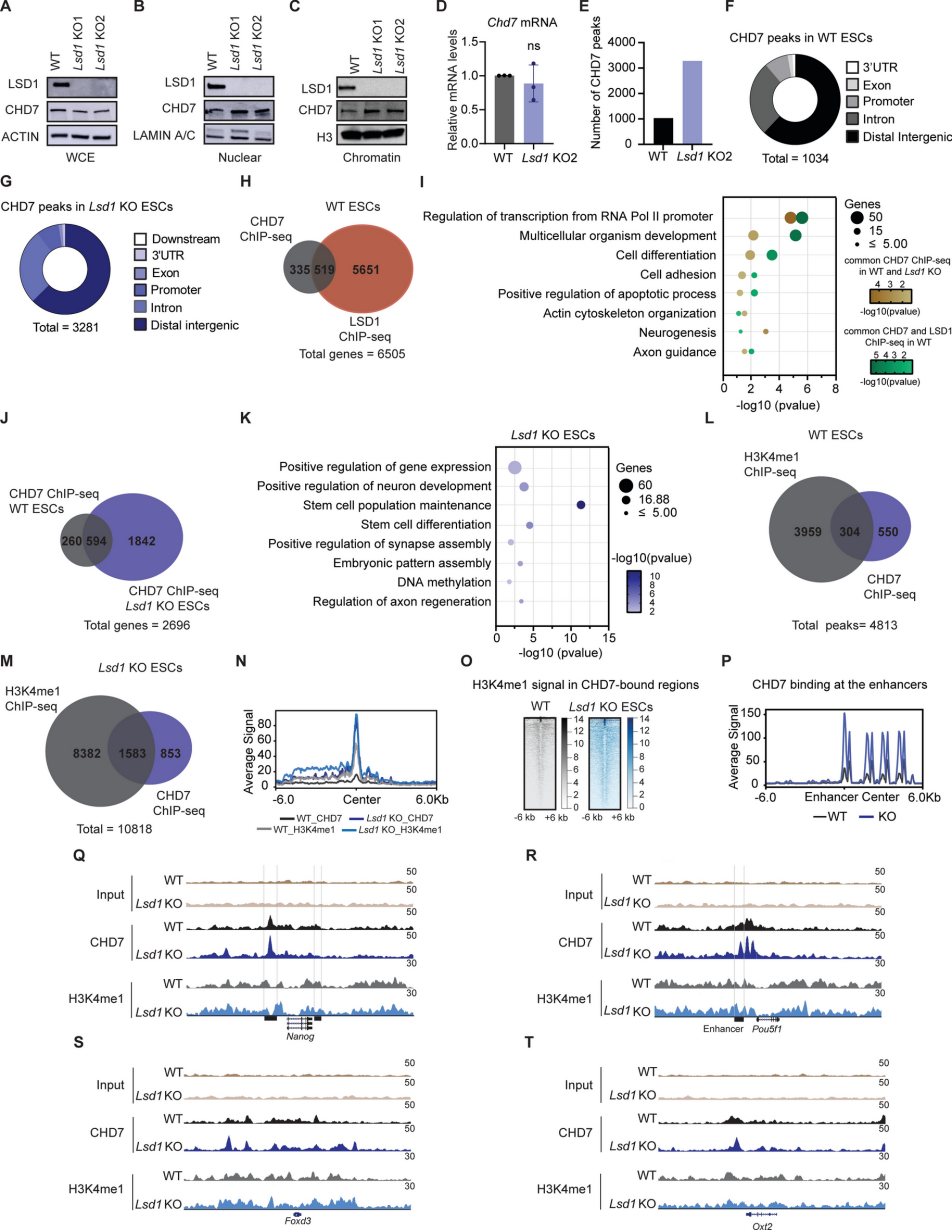
ChIP-seq analysis of CHD7 and LSD1. (**A–C**) WB of LSD1 and CHD7 in WCE, nuclear and chromatin fractions of WT and *Lsd1* KOs mouse ESCs. ACTIN, LAMIN A/C, and H3 were used as loading controls. The represented blots are from different gels of the same biological replicate. (**D**) Bar diagram representing RT-qPCR of *Chd7* in WT and *Lsd1* KO2 mouse ESCs. mRNA levels are relative to WT mouse ESCs. (**E**) Number of common CHD7 peaks retrieved from two independent biological replicates of CHD7-ChIP seq in WT and *Lsd1* KO mouse ESCs. (**F** and **G**) Genomic distribution of CHD7 binding in the promoter (within 5 kb upstream of TSS), distal intergenic, exon, UTR, downstream and intron in (**F**) WT and (**G**) *Lsd1* KO mouse ESCs. (**H**) Venn diagram of overlapped genes between CHD7 and LSD1 ChIP-seq in WT mouse ESCs. (**I**) GO analysis of biological processes of genes associated with CHD7 in WT and *Lsd1* KO mouse ESCs (brown) and common LSD1 and CHD7 peaks in WT mouse ESCs (green). (**J**) Venn diagram depicting the genes identified from CHD7 ChIP in WT and *Lsd1* KO2 mouse ESCs. (**K**) GO analysis of biological processes of genes associated with CHD7 ChIP in *Lsd1* mouse ESCs. (**L** and **M**) Venn diagram representing overlapped genes between H3K4me1 and CHD7 ChIP-seq in (L) WT and (M) *Lsd1* KO2 mouse ESCs. (**N**) Average signal of H3K4me1 and CHD7 ChIP-seq in co-occupied regions in WT and *Lsd1* KO2 mouse ESCs. (**O**) Density plot representing H3K4me1 binding in CHD7 peaks (common with WT and unique to *Lsd1* KO2) upon deletion of *Lsd1* in mouse ESCs. (**P**) Occupancy of CHD7 at the enhancers in WT and *Lsd1* KO2 mouse ESCs. (**Q-T**) CHD7 and H3K4me1 ChIP-seq signals in WT and *Lsd1* KO2 mouse ESCs at the (**Q**) *Nanog*, (**R**) *Pou5f1* (**S**) *Foxd3* and (**T**) *Otx2* genomic regions. Respective inputs are depicted in grey. Enhancers for *Nanog* and *Pou5f1* are marked as black squares. Data are represented as mean ± SD, and each experiment was performed with n = 3 (**D**) and n = 2 (**E**) replicates. *P < 0.05, **P < 0.01, ***P < 0.001 and ****P < 0.0001. Data in (**D**) were analyzed using an unpaired Student’s *t*-test. Results are one representative of n = 3 independent biological experiments (**A**–**C**).

To investigate the interplay between CHD7 and LSD1, we checked CHD7 protein expression in whole cell extracts (WCE), nuclear and chromatin fractions of WT and *Lsd1* KO mouse ESCs. We observed a modest decrease in the total CHD7 protein levels after *Lsd1* loss; however, the expression of CHD7 was increased in the nuclear and chromatin extracts of WT and *Lsd1* KO mouse ESCs. Such an increase of CHD7 in nuclear and chromatin fractions was not associated with *Chd7* mRNA levels, suggesting that deletion of *Lsd1* promotes CHD7 chromatin recruitment in mouse ESCs (Fig. 2A–D). Given that both CHD7 and LSD1 preferentially bind to active enhancers, we performed chromatin immunoprecipitation with massively high-throughput sequencing (ChIP-seq) of CHD7 in WT and *Lsd1* KO mouse ESCs. Consistent with elevated levels of CHD7 in chromatin upon *Lsd1* deletion, we identified 3281 peaks of CHD7 in *Lsd1* KO mouse ESCs, whereas 1034 peaks were identified in WT mouse ESCs (Fig. 2E; Table S2). These peaks are distributed along the genome, preferentially enriched at distal regions in both WT and *Lsd1* KO mouse ESCs (Fig. 2F and G). Further, to explore whether LSD1 and CHD7 bind to the same genomic regions, we intersect CHD7 ChIP-seq data with LSD1 ChIP-seq data in WT mouse ESCs from our previous study^15^ and we found ~ 60.7% of CHD7 binding sites co-bound by LSD1 in WT ESCs (Fig. 2H). GO analysis for biological processes of CHD7-bound regions and co-bound regions by both CHD7 and LSD1 indicated that they bind to genes related to “transcription”, “multicellular development”, and "neuron-related categories" (Fig. 2I), indicating a potential interplay between CHD7 and LSD1 in the regulation of cellular differentiation. Next, we overlapped genes bound by CHD7 in WT and *Lsd1* KO mouse ESCs and found that ~ 69.5% of the genes in WT ESCs were common with *Lsd1* KO ESCs (Fig. 2J). The subsets of genes exclusively bound by CHD7 in *Lsd1* KO mouse ESCs (1842) were enriched for biological processes such as "stem cell maintenance", “DNA methylation”, “axon regeneration”, among others (Fig. 2K).

It has been previously shown that *Lsd1* ablation leads to a global accumulation of H3K4me1^15^ and that CHD7 preferentially localizes to chromatin regions enriched with H3K4me1^19,20^. To investigate whether CHD7 recruitment to chromatin in *Lsd1* KO ESCs is linked to H3K4me1 deposition, we integrated publicly available H3K4me1 and CHD7 ChIP-seq data from WT and *Lsd1* KO mouse ESCs^15^. In WT ESCs, 34.15% of genes were co-occupied by CHD7 and H3K4me1, which increased to 64.49% in *Lsd1* KO ESCs (Fig. 2L and M). Further, analysis of these co-occupied regions revealed a significant increase in the overlap between H3K4me1 and CHD7 at the center of peaks following *Lsd1* deletion (Fig. 2N).

Additionally, we examined the distribution of H3K4me1 at CHD7 binding regions, including those common between WT and *Lsd1* KO, as well as those unique to *Lsd1* KO ESCs. We observed a heightened H3K4me1 signal in CHD7-bound regions in *Lsd1* KO ESCs compared to WT (Fig. 2O). Moreover, there was increased CHD7 co-occupancy, particularly at the center and downstream regions of enhancers (Fig. 2P). Indeed, loci regulating self-renewal (e.g., *Nanog* and *Pou5f1*) and neurogenesis (e.g., *Otx2* and *Foxd3*) exhibited increased H3K4me1 marks with elevated CHD7 occupancy in *Lsd1* KO mouse ESCs compared to WT mouse ESCs. Specifically, enhancers of *Pou5f1* and *Nanog* showed heightened co-occupancy of CHD7 and H3K4me1 following *Lsd1* deletion (Fig. 2Q–T). Altogether, these results suggest that increased H3K4me1 levels upon *Lsd1* deletion potentially promote CHD7 recruitment to chromatin, allowing it to bind to unique regulatory regions in *Lsd1* KO mouse ESCs.

### *Chd7* deletion does not exacerbate proliferative defects in *Lsd1* KO ESCs

**Fig. 3:**
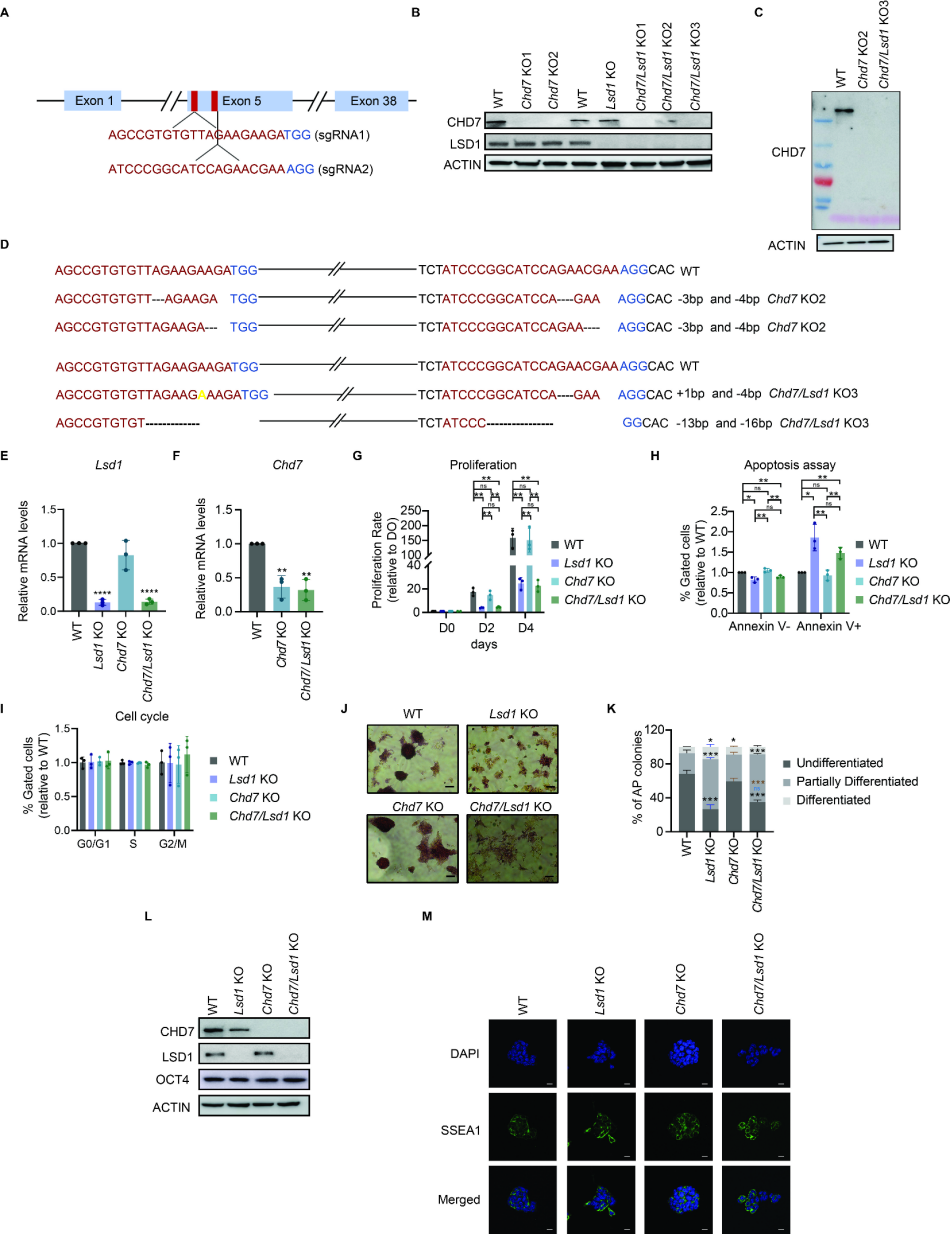
Assessment of the phenotype of *Chd7* and *Chd7/Lsd1* KO mouse ESCs. (**A**) Schematic representation of target sites in the genomic DNA of *Chd7*. The sgRNAs and PAM sequences are highlighted in red and blue, respectively. (**B**) Western blot of LSD1 and CHD7 on WCE from selected clones. ACTIN is used as loading control. The represented blots are from different gels. (**C**) Full western blot of CHD7 on WCE on WT, *Chd7* KO2 and *Chd7/Lsd1* KO3 mouse ESCs. ACTIN is used as a loading control. (**D**) Sanger sequencing analysis of *Chd7* KO2 (top panel) and *Chd7/Lsd1* KO3 mouse ESCs (bottom panel). Deletions and insertions are represented as dashes and in yellow, respectively. (**E** and **F**) RT-qPCR of (**E**) *Lsd1* in WT, *Lsd1* KO, *Chd7* KO, and *Chd7/Lsd1* KO ESCs and (**F**) *Chd7* in WT, *Chd7* KO and *Chd7/Lsd1* KO mouse ESCs. mRNA levels are relative to WT mouse ESCs. (**G**) Proliferation rate of WT, *Lsd1* KO*, Chd7* KO*,* and *Chd7/Lsd1* KO mouse ESCs at indicated time points relative to day 0. (**H**) Percentages of live (Annexin V-) and apoptotic cells (Annexin V +) in WT, *Lsd1* KO*, Chd7* KO*,* and *Chd7/Lsd1* KO mouse ESCs. (**I**) Bar diagram depicting the percentages of cells relative to WT at G0/G1, S, and G2/M phases in *Lsd1* KO, *Chd7* KO, *Chd7/Lsd1* KO mouse ESCs. (**J**) AP staining of WT, *Lsd1* KO, *Chd7* KO, and *Chd7/Lsd1* KO mouse ESCs and (**K**) Percentages of undifferentiated (UD), partially differentiated (PD), and differentiated (D) colonies from cells analyzed in (**K**). Scale bars: 20 μm. (**L**) Western blots of LSD1, CHD7 and OCT4 on the WCE of WT, *Lsd1* KO, *Chd7* KO and *Chd7/Lsd1* KO mouse ESCs. β-ACTIN is used as the loading control. (**M**) Representative immunofluorescence images of SSEA1 in WT, *Lsd1* KO, *Chd7* KO and *Chd7/Lsd1* KO mouse ESCs. DAPI was used as the nuclear marker. Scale bars, 20 μm. Data are represented as mean ± SD, and each experiment was performed with n = 3 replicates. ns- non-significant, *P < 0.05, **P < 0.01, ***P < 0.001 and **** *P* < 0.0001. Data in (**G**, **H**, and **K**) were analyzed using an unpaired Student’s *t*-test, (**E**, **F** and **I** (ns)) analyzed with two-way ANOVA. Each dot in the bar graphs represents independent biological replicates. Statistical comparison for (**G**, **H** and **I**): WT vs *Lsd1* KO, *Chd7* KO and *Chd7/Lsd1* KO; *Lsd1* KO vs *Chd7* KO and *Chd7/Lsd1* KO; and *Chd7* KO vs *Chd7/Lsd1* KO mouse ESCs. Statistical comparison for K (WT vs *Lsd1* KO, *Chd7* KO and *Chd7/Lsd1* KO is represented in black, *Lsd1* KO vs *Chd7* KO and *Chd7/Lsd1* KO in blue and *Chd7* KO vs *Chd7/Lsd1* KO mouse ESCs in brown, respectively). Results are one representative of n = 3 independent biological replicates (**C**, **J**, **L** and **M**).

To further investigate the role of CHD7 in stem cell pluripotency and differentiation, we deleted *Chd7* in both WT and in *Lsd1* KO mouse ESCs, with the *Lsd1* KO lines having been originally generated in our previous study^15^. We targeted exon 5 of *Chd7* employing two distinct single guide RNAs (sgRNAs). Specifically, sgRNA1 was designed to target the start codon of *Chd7,* whereas the sgRNA2 targeted a region 500 base pairs (bp) downstream of sgRNA1 (Fig. 3A). The absence of CHD7 protein following *Chd7* gene targeting was confirmed by western blotting (Fig. 3B, C). From these clones, we selected *Chd7* KO2 and *Chd7/Lsd1* KO3 for further analysis, which will be referred to as *Chd7* KO and *Chd7/Lsd1* KO, respectively. Sanger sequencing revealed two different insertion/deletions (indels) (− 3 and − 4 bp) in *Chd7* KO2, whereas four different indels (+ 1 bp, − 4 bp, − 13 bp, and − 16 bp) were detected in *Chd7/Lsd1* KO3 mouse ESCs (Fig. 3D). *Chd7* KO and *Chd7/Lsd1* KO mouse ESCs were further validated by reverse transcription followed by quantitative PCR (RT-qPCR) (Fig. 3E and F).We assessed the phenotype of the engineered cell lines by performing proliferation, apoptosis, and cell cycle assays. We did not detect proliferation and apoptosis defects in *Chd7* KO mouse ESCs. However, similar to *Lsd1* KO, *Chd7/Lsd1* KO mouse ESCs showed a dramatically decreased proliferation with augmented apoptosis compared to WT and *Chd7* KO mouse ESCs (Figs. 3G and H). This result implied that the defective viability observed in the double KO arises predominantly from the deletion of *Lsd1* in mouse ESCs. Of note, no significant difference in the cell cycle profile was detected upon combined /single deletion of *Chd7* and *Lsd1* (Fig. 3I). We next sought to determine whether KO clones could remain pluripotent by assessing alkaline-phosphatase (AP) staining. *Chd7* KO ESCs exhibited a strong AP activity, similar to WT mouse ESCs. However, both *Chd7/Lsd1* and *Lsd1* KO mouse ESCs were organized as monolayers with undefined colony morphology (Fig. 3J). The quantification of AP-staining revealed an increased number of partially differentiated colonies in *Chd7/Lsd1* KO compared to WT and *Chd7* mouse ESCs, possibly due to generic growth defects occurring upon *Lsd1* ablation (Fig. 3K). However, these double KO clones did not show a significant difference in the expression of OCT4 and SSEA1 proteins compared to WT and *Chd7* KO mouse ESCs through western blotting and immunoassay staining, respectively (Fig. 3L and M). Overall, our data suggests that ablation of *Chd7* and *Lsd1* does not affect the pluripotency of ESCs; however, the combined deletion of *Chd7* and *Lsd1* exhibited growth defects that mirrored *Lsd1* KO phenotype.

### *Chd7* deletion leads to transcriptional dysregulation related to neuronal development

**Fig. 4:**
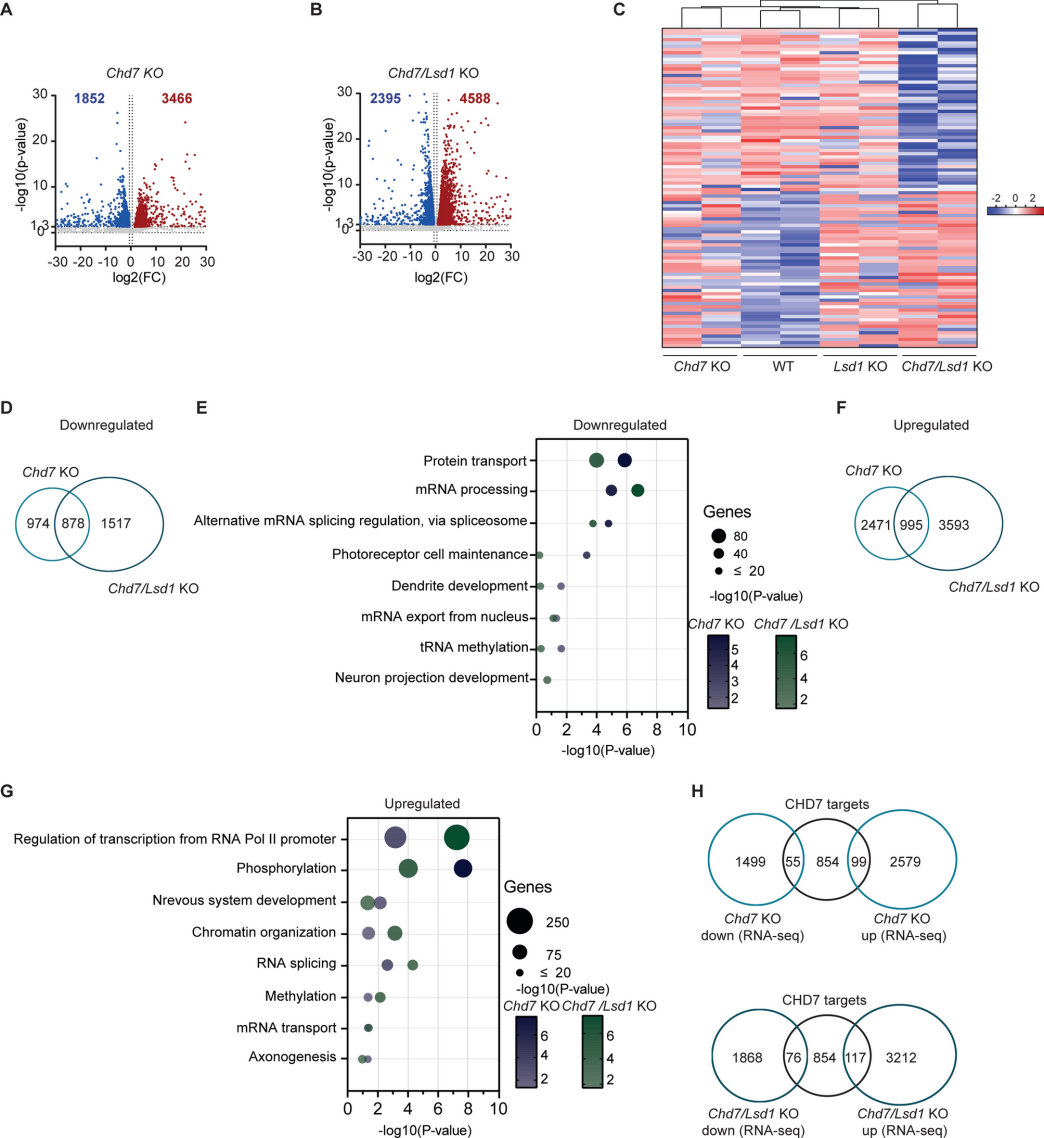
CHD7 regulates transcription of neuronal genes. (**A** and** B**) Volcano plots showing the distribution of differentially expressed transcripts in (**A**) *Chd7* KO and (**B**) *Chd7/Lsd1* KO compared to WT mouse ESCs. Red dots represent upregulated genes, blue dots represent down-regulated genes (*P* < 0.05; fold-change, FC > 1.5). (**C**) Heatmap depicting upregulated (red) and downregulated (blue) genes retrieved from the RNA-seq data of WT, *Lsd1* KO, *Chd7* KO and *Chd7/Lsd1* KO mouse ESCs. (**D**) Venn diagram of common downregulated transcripts between *Chd7* KO and *Chd7/Lsd1* KO ESCs. (**E**) Biological processes-based GO analysis of downregulated genes in *Chd7* KO and *Chd7/Lsd1* KO mouse ESCs. (**F**) Venn diagram of common upregulated transcript between *Chd7* KO and *Chd7/Lsd1* KO mouse ESCs. (**G**) Gene ontology analysis of biological processes related to the upregulated genes of *Chd7* KO and *Chd7/Lsd1* KO compared to WT mouse ESCs. (**H** and** I**) Overlap of RNA-seq data of (**H**) *Chd7* KO and (**I**) *Chd7/Lsd1* KO with CHD7 ChIP in WT mouse ESCs showing the downregulated and upregulated genes bound by CHD7. Downregulated and upregulated genes that are bound by CHD7 are depicted in blue and red, respectively.

To interrogate the impact of *Chd7* deletion on the ESCs transcriptome, we conducted RNA-seq analysis in WT, *Chd7* KO, and *Chd7*/*Lsd1* KO mouse ESCs (Figs. 4A and ; Table S3). Loss of *Chd7* resulted in 1852 downregulated transcripts, whereas combined *Lsd1* and *Chd7* ablation led to 2395 downregulated transcripts (fold change (FC) > 1.5; *P* < 0.05) (Fig. 4A and B). In contrast, *Chd7* KO and *Lsd1/Chd7* KO mouse ESCs exhibited 3466 and 4588 upregulated transcripts, respectively (FC > 1.5; *P* < 0.05) (Fig. 4A and B). We then generated a heatmap to visualize the expression patterns of differentially expressed genes in *Chd7* KO and *Chd7/Lsd1* KO mouse ESCs, integrating our RNA-seq data from *Lsd1* KO mouse ESCs as reported in our previous study^15^. Hierarchical clustering of the heatmap, based on the top differentially expressed genes in *Chd7/Lsd1* KO ESCs, revealed that the transcriptomes of *Lsd1* and *Chd7/Lsd1* KO mouse ESCs clustered more closely together than those of *Chd7 and Chd7/Lsd1* KO mouse ESCs (Fig. 4C).

Notably, ~ 47% of downregulated transcripts in *Chd7* KO were common with *Chd7/Lsd1* KO mouse ESCs (Fig. 4D). The enriched GO terms for these commonly downregulated genes included “protein transport”, “splicing”, and “tRNA methylation”, as well as categories related with ectoderm differentiation such as “dendrite development” and "neuronal projection development" (Fig. 4E). Indeed, prior studies have demonstrated that CHD7 remodels the chromatin accessibility to activate genes associated with the specification of neuronal identity and the transition of neuronal progenitors cells to mature neurons^29,30^. Likewise, LSD1 acts as a positive regulator of neuron differentiation and functions^31,32^. In contrast, only ~ 28% of upregulated genes in *Chd7* KO were similar to *Chd7/Lsd1* KO mouse ESCs (Fig. 4F). Analysis of upregulated genes in both *Chd7* KO and *Chd7/Lsd1* KO ESCs revealed enrichments related to "nervous system development", and “chromatin organization”, amongst others (Fig. 4G). Next, to evaluate the impact of co-occupancy of LSD1 and CHD7 on transcription regulation of their target genes, we overlapped the differentially expressed genes in *Chd7* and *Chd7/Lsd1* KO mouse ESCs with CHD7 ChIP-seq data. We found that ~ 18% and 22.59% of genes that were repressed or activated upon deletion of *Chd7*, and both *Chd7* and *Lsd1* were bound by CHD7 (Fig. 4H). Altogether, this data shows that CHD7 can act either as a repressor or activator of gene expression and that, although *Chd7/Lsd1* double ablation leads to a more profound effect in transcriptional changes, loss of *Chd7* possesses a unique regulatory signature.

### Enhanced impairment of EB formation with combined *Chd7* and *Lsd1* ablation

**Fig. 5:**
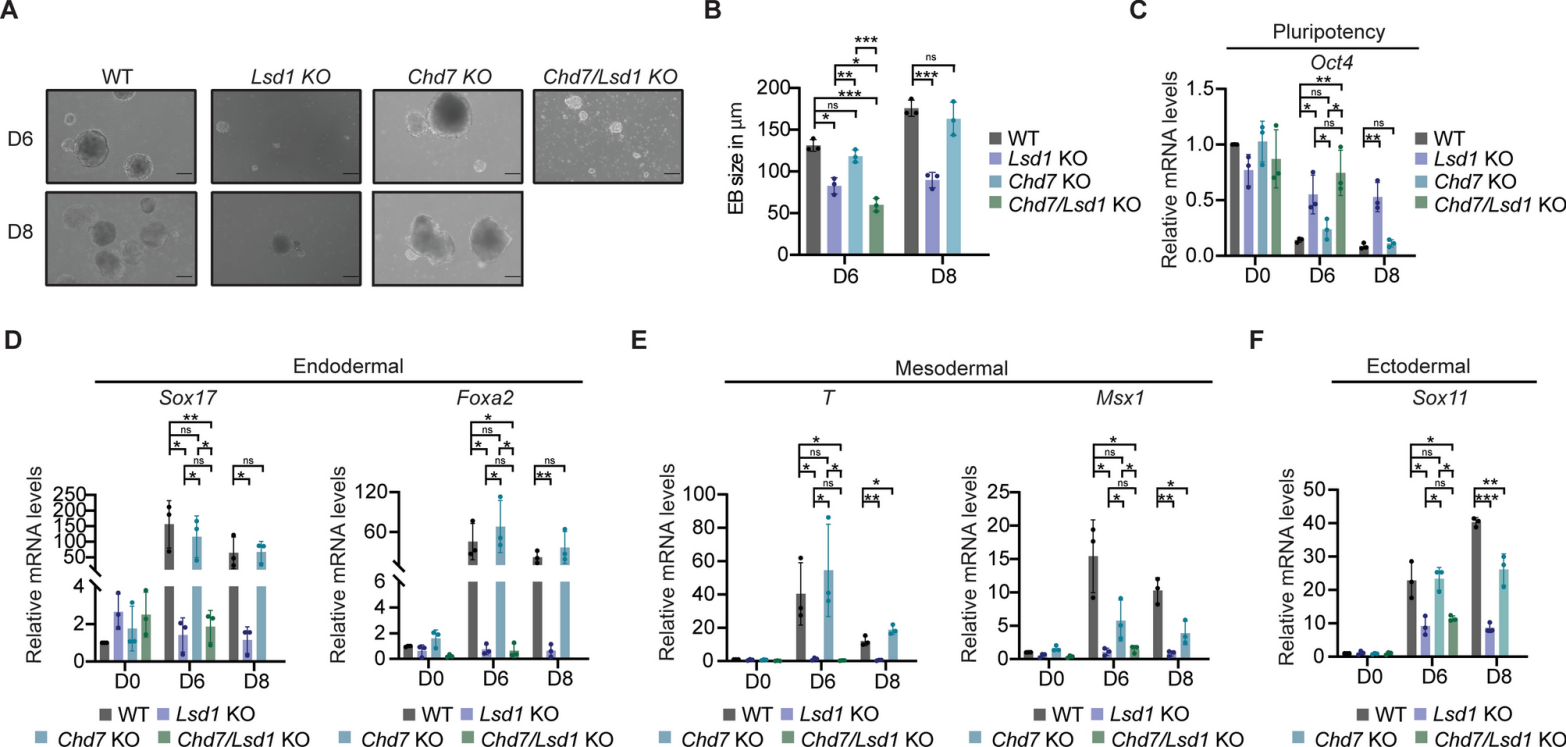
CHD7 is dispensable for EB differentiation. (**A**) Representative brightfield images of EBs and (**B**) measurement of EBs size derived from WT, *Lsd1* KO, *Chd7* KO*,* and *Chd7/Lsd1* KO mouse ESCs at the indicated time points. Scale bars: 200 µm. (**C**–**F**) RT-qPCR analysis of (**C**) pluripotency (*Oct4*), (**D**) endodermal (*Sox17* and *Foxa2*), (**E**) mesodermal (*T* and *Msx1*), and (**F**) ectodermal (*Sox11*) markers in the EBs generated from WT, *Lsd1* KO, *Chd7* KO, and *Chd7/Lsd1* KO mouse ESCs at the indicated days after differentiation. mRNA levels are relative to the expression of WT mouse ESCs at day D0. Data are represented as mean ± SD. *P < 0.05, **P < 0.01, and ***P < 0.001. Data in (**B**–**F**) were analyzed using an unpaired Student’s *t*-test. Each dot in the bar graphs represents independent biological replicates. Statistical comparison for (**C**–**F**): Day 6 (WT vs *Lsd1* KO, *Chd7* KO and *Chd7/Lsd1* KO; *Lsd1* KO vs *Chd7* KO and *Chd7/Lsd1* KO; *Chd7* KO vs *Lsd1* KO *and Chd7/Lsd1* KO mouse ESCs) and Day 8 (WT vs *Lsd1* KO, and *Chd7* KO mouse ESCs).

To investigate the impact of double ablation of both *Chd7* and *Lsd1* on differentiation, we generated embryoid bodies (EBs) from WT, *Lsd1* KO, *Chd7* KO, and *Chd7/Lsd1* KO mouse ESCs. EBs derived from *Chd7* KO mouse ESCs displayed normal morphology and size (Fig. 5A and B). However, both *Lsd1* KO and *Chd7/Lsd1* KO EBs exhibited defective differentiation, and *Chd7/Lsd1* KO EBs being half the size of WT-derived EBs, did not survive beyond day 6 of differentiation (Fig. 5A and B), suggesting that the combined loss of both *Chd7* and *Lsd1* leads to more severe impairment in EB formation compared to the loss of *Lsd1* alone.

Further analysis of gene expression of pluripotency factors and lineage-specific markers revealed that *Chd7* KO EBs silenced *Oct4* expression and induced the expression of the endodermal markers *Sox17* and *Foxa2*, and the mesodermal factor *Brachyury* (*T*) similar to WT control cells (Fig. 5C–E). Yet, they failed to upregulate *Msx1* (mesoderm), and the expression of *Sox11* (ectodermal) was significantly lower than in WT-derived EBs (Figs. 5E, F), indicating that CHD7 is required for the selective induction of lineage-specific markers but not for the viability of EBs. In contrast, *Chd7/Lsd1* KO and *Lsd1* KO mouse EBs were unable to abolish *Oct4* expression (Fig. 5C) and showed significant downregulation of endodermal (*Sox17*, *Foxa2)*, mesodermal (*T*, *Msx1)*, and ectodermal (*Sox11)* markers compared to WT-derived EBs along the course of differentiation (Figs. 5D–F). This data suggests that while *Lsd1* depletion primary contributes to the impaired EB formation observed in *Chd7/Lsd1* KO EBs, the combined absence of both *Chd7* and *Lsd1* exacerbates this phenotype.

## Discussion

During development, the precise transition of cell fate relies on the intricate orchestration of gene expression, involving a complex interplay between transcriptional machinery and chromatin remodeling complexes. The dynamic modulation of chromatin accessibility is particularly critical for maintaining the identity of ESCs. However, the detailed understanding of how these regulatory mechanisms interact is still incomplete. Our study aims to elucidate the molecular interplay between two epigenetic regulators, LSD1 and CHD7, and to investigate how this interaction influences the balance between self-renewal and differentiation in ESCs.

Through our interactome analysis, we identified CHD7 as a novel interaction partner of LSD1 in mouse ESCs. While LSD1 is a H3K4 demethylase with diverse roles in ESC self-renewal and pluripotency^4,15^, CHD7 is a chromatin remodeler that binds to enhancers marked by H3K4me1^19^. Previous studies have shown that CHD7 can act as a rheostat in modulating ESC-specific gene expression. Loss of *Chd7* increases the transcription of certain highly expressed ESC-specific genes, implying an antagonistic function of CHD7 at enhancers^20^. However, the mechanism of how CHD7 regulates transcription is not fully elucidated yet. Our findings demonstrate that CHD7 interacts with LSD1 via its helicase and BRK domains, suggesting a potential role for this interaction in nucleosome remodeling.

Previous studies have demonstrated that CHD7 is associated with a group of active enhancers bearing H3K4me1, a crucial mark that facilitates the binding of pivotal chromatin factors^33^. Our data show that while CHD7 binds to chromatin regions enriched in H3K4me1, deletion of *Lsd1* significantly enhances the recruitment of CHD7 to chromatin. This increased binding could be a consequence of the gain of H3K4me1 at enhancer regions upon loss of *Lsd1* in mouse ESCs^15^. CHD7 binds mainly to distal intergenic regions, and a subset of genes co-bound by CHD7 and LSD1 are associated with neuronal differentiation. Specifically, CHD7-unique binding sites are related to the establishment of functional neuronal circuits, such as synapse assembly and axon regeneration, suggesting the stage-specific role of CHD7-mediated chromatin remodeling during axon maturation. We cannot rule out the possibility that LSD1 and CHD7 could either compete or cooperate in the differentiation of certain neuronal subtypes, which could impact the proper length and branching of neurites^30^.

Our study further explores the impact of *Chd7* and *Lsd1* deletion on mouse ESCs. While *Chd7* KO mouse ESCs exhibit normal proliferation and form typical round colonies similar to WT mouse ESCs, the combined ablation of both *Chd7* and *Lsd1* results in severe growth defects. This dual knockout scenario leads to an increase in partially differentiated ESC colonies and triggers apoptosis, resembling the phenotype observed in *Lsd1* KO mouse ESCs. These results indicate that CHD7 may not be critical for ESC self-renewal, consistent with previous studies on *Chd7*-null ESCs, which showed that despite CHD7’s association with master regulators such as OCT4 and NANOG, it is not essential for ESC self-renewal and pluripotency^20^. While CHD7 may modulate chromatin interactions, deletion of *Chd7* alone had a minimal impact on ESCs, implying its role in ESCs is secondary to LSD1.

Furthermore, the abrogation of *Chd7* and *Lsd1* in mouse ESCs results in the differential expression of genes related to nervous system development in mouse ESCs. CHD7 is highly expressed in dividing neural stem cells and progenitors within adult brain subventricular and subgranular zones. Deletion of *Chd7* in mice leads to hindered neurogenesis and aberrant dendritic growth^30,34^. In addition, CHD7 selectively coordinates the expression of key developmental genes such as *Otx1*, *Msx1* and *Sox2,* which are crucial for vestibular morphogenesis^35^. Mutations in *CHD7* are associated with CHARGE syndrome, underlining its role in human neurological and developmental disorders^36^. Similarly, LSD1 orchestrates gene expression programs associated with neurogenesis, such as postmitotic, fate-committed neurons, and photoreceptor differentiation^37–39^. We noted that *Chd7* KO ESCs were able to differentiate normally, albeit with a modest reduction in mesodermal (*Msx1*) and ectodermal (*Sox11*) markers upon EB differentiation. However, the double knockout of *Chd7* and *Lsd1* exhibited abnormal EB size and impaired upregulation of differentiation markers, in contrast to WT and *Chd7* KO mouse ESCs. Both the double knockout and *Lsd1* KO mouse ESCs showed defective differentiation and maintained high *Oct4* levels. Notably, the double knockout exhibited earlier lethality by day 6, while *Lsd1* KO mouse ESCs could be cultured for a longer period. This data suggests that while CHD7 intensifies the phenotypes resulting from *Lsd1* loss, it is not itself required for ESC self-renewal or differentiation. In summary, our study identifies CHD7 as a novel interactor of LSD1 and demonstrates that LSD1 and CHD7 co-occupy regulatory sites of a subset of genes related to cell differentiation and neurogenesis. Deletion of *Chd7* and *Lsd1* leads to severe differentiation defects, primarily driven by defects observed upon deletion of *Lsd1.* While CHD7 contributes to chromatin interactions, its role appears more context-dependent and secondary, fine-tuning chromatin accessibility. This highlights the importance of their co-occupancy on chromatin regulatory elements for precise gene expression during differentiation.

## Material and methods

### Antibodies

The following commercially available antibodies were used for western blot: anti-LSD1 (Abcam, ab17721, 1:2500), anti-βACTIN (Sigma, A5441, 1:5000), anti-H3 (Abcam, ab8895, 1:8000), anti-CHD7 (Cell Signaling, 6505, 1:2500), anti-FLAG (Sigma, F3165, 1:4000), anti-MYC (Cell Signaling, 2276S, 1:4000) and anti-Rabbit-HRP (Thermo Scientific, A-11011, 1:8000), anti-Mouse-HRP (Thermo Scientific, A11029,1:1000). ChIP analysis was performed with anti-CHD7 (Cell Signaling, 6505). Co-IP experiments were performed with anti-MYC (Cell Signaling, 2276S, 2 µg), anti-LSD1 (Abcam, ab17721, 1:2500), and anti-CHD7 (Cell Signaling, 6505, 1:2500) antibodies.

### Cell culture

CCE murine ESCs were cultured on 0.1% gelatin-coated tissue culture plates under feeder-free culture conditions. The culture medium comprised Dulbecco’s modified Eagle’s medium (DMEM) high glucose, 15% fetal bovine serum (FBS, Gibco), 1% MEM non-essential amino acids (Sigma-Aldrich), 0.1 mM of β-mercaptoethanol, 1% L-glutamine (Hyclone) and 1% penicillin/streptomycin (Gibco) and Leukemia inhibitory factor (LIF, R&D systems). Cells were maintained at 37 °C with 5% CO_2_.

### EB differentiation assays

ESCs were plated at a density of 8.8 × 10^4^ cells/cm^2^ in low attachment conditions in a complete medium without LIF. EBs were harvested for extraction of total RNA at the indicated time points. The medium was changed every 48 h. Sizes of EB were quantified using NIS-elements software.

### Generation of CRISPR/Cas9 KO mouse ESCs

ESCs were seeded at a density of 8 × 10^3^ cells/well in a 12 well-plate. After 24 h of seeding, cells were transfected with 0.8 µg of Cas9 expression vector PX459 (Addgene, #62,988) containing the corresponding cloned sgRNAs using Lipofectamine (Invitrogen) following the manufacturer’s instructions. All sgRNA-Cas9 plasmids were prepared by ligation (T7 DNA ligase, Fermentas) of annealed complementary oligonucleotides with the PX459 vector digested with BbsI (BpilI) (Thermo Scientific). KO clones were screened using PCR and validated with western blot and Sanger sequencing. All sgRNAs were designed using Zhang lab’s online tool (https://www.zlab.bio/resources). sgRNAs and primers sequences are indicated in Table S4.

### Alkaline phosphatase (AP) activity

An alkaline phosphatase (AP) staining kit (Stemgent) was utilized to detect AP activity following the manufacturer’s instructions.

### RT-qPCR analysis

Total RNA was extracted using the RNeasy Mini Kit (Qiagen) and subjected to RT-qPCR. Briefly, 1 μg of total RNA was reverse transcribed using the RevertAid First Strand cDNA Synthesis kit (Invitrogen). Quantitative PCR (qPCR) was performed using the Power Up SYBR Green qPCR Master Mix (Applied Biosystems). Primers sequences used for this study are listed in Table S4.

### RNA-seq library preparation

RNA-Seq library preparation was performed at Novogene facilities. Samples were sequenced by the Illumina HiSeq™ platform (Illumina) as 100 bp pair-ended reads.

### RNA-seq analysis

FASTQ RNA-Seq reads were filtered by quality and pseudoaligned to mouse genome reference database (mm10). Differentially expressed genes were identified by considering a binomial distribution in MATLAB and using a false discovery rate (FDR) < 0.005 and fold-change > 1.5.

### ChIP-seq

Chromatin immunoprecipitations were performed according to our prior study’s protocol^40^. Mouse ESCs were chemically crosslinked by adding 1/10 volume of fresh 11% formaldehyde solution for 10 min at RT, followed by quenching with 1/20 volume of 2.5 M of glycine for 5 min at RT. Cells were then washed twice with ice-cold PBS, scraped, and collected by centrifugation. The cell pellet underwent lysis the lysis buffer 1 (50 mM, Hepes–KOH pH 7.5, 140 mM NaCl, 1 mM EDTA, glycerol (10% vol/vol) NP-40 (0.5% vol/vol), Triton X-100 (0.25% vol/vol)) for 10 min at 4 °C , followed by second lysis in buffer 2 (200 mM NaCl, 1 mM EDTA, 0.5 mM EGTA, 10 mM Tris (pH 8)) with complete protease inhibitors. Subsequently, cells were sonicated in lysis buffer 3 (100 mM NaCl, 1 mM EDTA, 0.5 mM EGTA, 10 mM Tris pH 8, Na-Deoxycholate (DOC) (0.1% vol/vol), N-lauroyl sarcosine (0.5% vol/vol) for 16 × 30-s pulses (30-s pause between pulses) at high power in a Bioruptor® Sonication System (Diagenode), followed by centrifugation at high speed for 15 min. The supernatant containing the chromatin fraction was then subjected to immunoprecipitation.

A spike-in control using the human MCF10A cell line was employed to normalize ChIP-seq reads. For this purpose, human MCF10A chromatin was prepared, as described previously. 5% of MCF10A (vol/vol) chromatin was added to the chromatin and antibodies immunoprecipitation complex. This mixture was then combined 50 μl of Protein G magnetic beads and incubated overnight at 4 °C. Protein-DNA bead complexes underwent five washes with RIPA buffer (50 mM Hepes pH 7.6, 1 mM EDTA, DOC (0.7% vol/vol), NP-40 (1% vol/vol), 0.5 M LiCl), followed by washing with TE containing 50 mM NaCl. Subsequently, the protein-DNA complexes were eluted from the beads twice by incubating with 100 μL of elution buffer (50 mM Tris pH 8, 10 mM EDTA, SDS (1% vol/vol)) at 65 °C for 15 min with shaking. Reverse crosslinking was achieved by adding 200 mM NaCl in the eluate and incubating it at 65 °C overnight with shaking. RNA and protein were eliminated from the samples by treating with RNase A and proteinase K as the manufacturer’s recommendations. DNA was subsequently purified by phenol–chloroform extraction, followed by ethanol precipitation. The input sample was also treated for crosslink reversal and following steps after this.

Library construction and paired-end read sequencing (20 M per sample) were performed using Illumina technology at Novogene (United Kingdom). Raw reads were aligned to the human (hg38) and mouse (mm10) genomes using BWA^41^. The alignments were normalized using the number of reads uniquely mapped to the human genome^42^. Peaks were then called using MACS2^43^ with pooled IPs and inputs (FDR < 0.05), and they were annotated using the ChIPseeker^44^ package in R. Subsequent analyses were performed using bedtools^45^ for peak overlapping and MATLAB, GbiB^46^, and EaSEQ^47^ for visualization.

### Cellular proliferation

2 × 10^4^ cells were seeded into each well of a 6-well plate, and subsequently counted every other day for a duration of 4 days using trypan blue (BioRad)

### Apoptosis assay and cell cycle analysis

Apoptosis assay and cell cycle analysis were performed using Muse™ Cell Analyzer from Millipore following the manufacturer’s instructions.

### Nuclear extractions

Cells were washed with cold 1X PBS twice and pelleted. The pellet was then suspended in at least 5 volumes of buffer A (10 mM HEPES pH 7.9, 1.5 mM MgCl_2_, 10 mM KCl, 2 mM DTT) in the presence of protease inhibitors (Fisher Scientific) and incubated for 10 min on ice. Following centrifugation, the pellet was resuspended in 2 volumes of buffer A, dounce-homogenized 10 times, and centrifuged at maximum speed for 15 min. The resulting nuclei pellets were resuspended in 2 volumes of buffer B (20 mM HEPES pH 7.9, 1.5 mM MgCl_2_, 500 mM NaCl, 25% Glycerol, 0.5 mM EDTA, 1 mM DTT) supplemented with protease inhibitors and incubated for 30 min on a rotator at 4 °C. Subsequently, the mixture was centrifuged at maximum speed for 20 min and the supernatant was frozen at − 80 °C for further analysis.

### Co-immunoprecipitation and immunoblotting

1 mg of nuclear extracts were pre-cleared with protein G magnetic beads (BioRad) for 1 h at 4 °C and incubated with specific antibodies overnight on a rotator at 4 °C. Following this, protein G magnetic beads were added to the lysate and antibody mix for 3 h before washing 4 times in ice-cold IP buffer (10 mM Tris pH 7.4, 1 mM EDTA, 1 mM EGTA; pH 8.0, 150 mM NaCl, 1% Triton X-100, 0.2 mM sodium orthovanadate) supplemented with protease inhibitors. Immunoprecipitated complexes were separated by SDS-PAGE, transferred to PVDF membranes (Invitrogen), and immunoblotted with the indicated antibodies followed by ECL detection (Thermo Scientific).

### Mass spectrometry analysis to identify LSD1-associated protein complexes

10 μg of LSD1 antibody were conjugated to 1 mg of Dynabeads® M-270 Epoxy following the manufacturer’s instructions (Invitrogen). Then, 1 mg of nuclear extracts were incubated with antibody-coupled beads overnight at 4 °C. After washing 4 times with NP-40 buffer (150 mM NaCl, 1% NP-40, 50 mM Tris–HCl pH 8), immunoprecipitated proteins were eluted with 100 μl of 200 mM Glycine buffer (pH 2.6) for 10 min at RT and neutralized by adding 1/10 volume of 1 M Tris–HCl pH 8. The immunoprecipitated protein complexes were subjected to LC–MS/MS analysis (SciLife Lab, Sweden).

### Statistical analysis

All values were expressed as mean ± SD. GraphPad Prism 9.00 for Windows was used to perform the statistical analysis (GraphPad Software, La Jolla California USA, www.graphpad.com). Statistical analysis was performed by the Unpaired Student’s t-test, and two-way ANOVA. A probability value of *P* < 0.05 was considered statistically significant.

## Supplementary Information

Supplementary Information 1.

Supplementary Information 2.

Supplementary Information 3.

Supplementary Information 4.

Supplementary Information 5.

Supplementary Information 6.

## Data Availability

All next-generation sequencing data is publicly accessed in Bioproject database (Submission ID: SUB140770796 and BioProject ID: PRJNA1172994).
