# Supplementary figures and images for: Cooperative role of LSD1 and CHD7 in regulating differentiation of mouse embryonic stem cells
Source: Sci Rep. 2024 Nov 18;14:28495. doi: 10.1038/s41598-024-78920-3 (PMC11574112; doi:10.1038/s41598-024-78920-3)

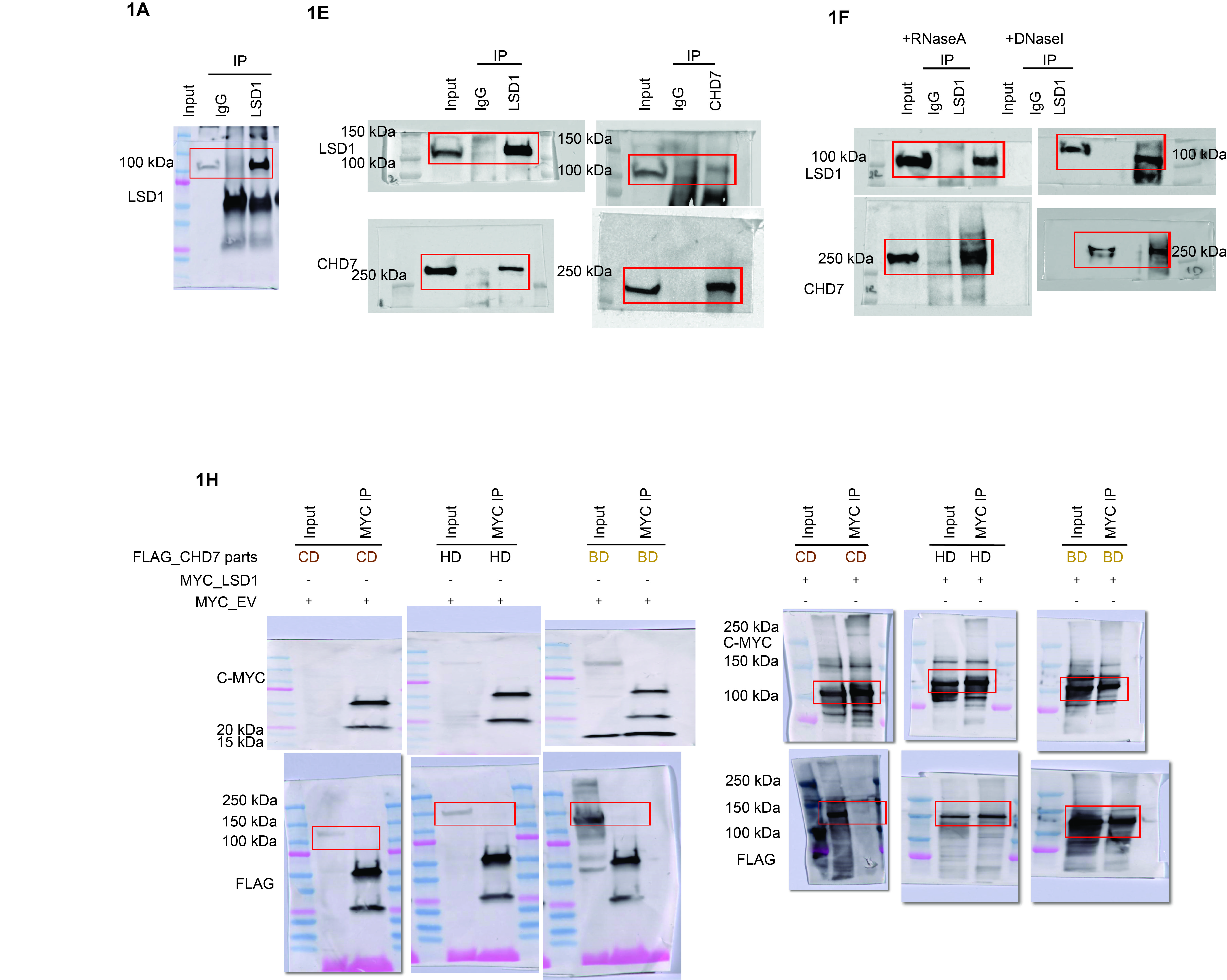

Supplement: Supplementary file 1 — Supplementary Information 1. [file 41598_2024_78920_MOESM1_ESM.jpg]
