# Supplementary material for: Cooperative role of LSD1 and CHD7 in regulating differentiation of mouse embryonic stem cells
Source: Sci Rep. 2024 Nov 18;14:28495. doi: 10.1038/s41598-024-78920-3 (PMC11574112; doi:10.1038/s41598-024-78920-3)
Supplement: Supplementary file 6 — Supplementary Information 6. [file 41598_2024_78920_MOESM6_ESM.docx]

**Table S4**. Sequence of primers and sgRNAs used in this study.

| **sgRNA** | | |
| --- | --- | --- |
| **Target gene** | **sgRNA** | **Sequence 5'-3'** |
| *Chd7* | sgRNA1 | CACCGAGCCGTTGTTAGAAGAAGA |
| *Chd7* | sgRNA2 | CACCGATCCCGGCATCCAGAACGAA |

| **qPCR** | | |
| --- | --- | --- |
| **Target gene** | **Forward 5'🡪3'** | **Reverse 5'🡪3'** |
| *Chd7* | CAGGAGGAAAAACGTGGAGGG | TCAGCATCCTCTGCACTTAACG |
| *Lsd1* | CACAGCAGTCCCCAAGTATGT | GCCTCTGCTGTCAAACTAGGA |
| *Oct4* | AGTTGGCGTGGAGACTTTGC | CAGGGCTTTCATGTCCTGG |
| *Nanog* | TCTTCCTGGTCCCCACAGTTT | GCAAGAATAGTTCTCGGGATGAA |
| *Sox17* | ACGCTCCAGTCTCGGACTAT | GCCGTAGTACAGGTGCAGAG |
| *Foxa2* | CCCTACGCCAACATGAACTCG | GTTCTGCCGGTAGAAAGGGA |
| *T* | GCTTCAAGGAGCTAACTAACGAG | CCAGCAAGAAAGAGTACATGGC |
| *Msx1* | TCATGGCCGATCACAGGAAG | CGACTGAGAAATGGCCGAGA |
| *Sox11* | ACGACCTCATGTTCGACCTGAGCT | CACCAGCGACAGGGACAGGTTC |
